# Medical teachers’ discursive positioning of doctors in relation to patients

**DOI:** 10.1111/medu.14074

**Published:** 2020-04-06

**Authors:** Tim Dornan, Selina Roy Bentley, Martina Kelly

**Affiliations:** ^1^ Centre for Medical Education Queen’s University Belfast Belfast UK; ^2^ Department of Education Development and Research Maastricht University Maastricht the Netherlands; ^3^ Department of Family Medicine Cumming School of Medicine University of Calgary Calgary Alberta Canada

## Abstract

**Context:**

An important part of a doctor’s identity is the social position he or she adopts relative to patients. Dialogic theory predicts that medical school discourses influence the positions students incorporate into their professional identities. As this may affect how students later exercise power in doctor‐patient relationships, we set out to examine how medical teachers position doctors in relation to patients.

**Methods:**

Informed by Holland’s Figured Worlds theory, which draws important assumptions from Bakhtin, we chose dialogic research methodology to examine how educators’ language positions doctors and may influence students’ identity formation. We recruited a maximum variation sample of 10 teaching staff and used open prompts in individual semi‐structured interviews to elicit discourses of doctors’ social position. We used Sullivan’s dialogic methodology reflexively to identify informative speech acts (utterances) and to examine how the language used in these constructed doctors’ positions.

**Results:**

Dominant discourses of Social Superiority, Technical Effectiveness, and Benevolence elevated doctors’ positions based on their social status, applied knowledge and trustworthiness, respectively. These positions were defended by predicating medical care on doctors’ mastery of treatments and their superior knowledge. A non‐dominant discourse of Distributed Power and Responsibility narrowed the positional gap by constructing doctors as empowering patients.

**Conclusions:**

Whereas three conservative discourses upheld doctors’ elevated social position, a non‐dominant, transformative discourse distributed power. We suggest that doctors will form the best relationships with patients when they are aware of these discourses and know how to navigate them. In pursuit of effective and compassionate patient care, we commend critical pedagogy as a means of articulating non‐dominant discourses and increasing students’, educators’ and doctors’ awareness of how they learn the positions of doctors.

## Abstract 2

Dornan et al. identify four discourses that position doctors in relationships with patients: superiority, effectiveness, benevolence, distribution. Awareness of these discourses could encourage students, faculty to better understand how status, power influence healthcare.

**Key Message**

Encouraging students to be critical of teachers' language could encourage them to form sharing relationships rather than adopt elevated positions over patients.

## 1 INTRODUCTION

Doctors have an elevated social position because possessing a medical degree empowers them to make decisions, share information and perform procedures that affect patients’ lives.^1^ Becoming ill, by contrast, deprives patients of powers they may have taken for granted, such as to work, to care for themselves, and to care for others. This lowers patients’ social position and places them in double jeopardy because they need power, as well as health, to ‘formulate their values, articulate and achieve their health needs, and fulfil their responsibilities’.^1^ Some doctors invest their power in empowering patients^2^ by actively seeking to narrow the gap between their own social position and the patient’s position for optimum clinical benefit.^3^ Others are unwilling to do this because holding an elevated position is an important part of their identity.^4^, ^5^ Social positioning, these arguments suggest, influences doctors’ provision of clinical care.

Medical students have less elevated social positions than doctors. In formal education settings, medical teachers may accentuate this by ‘pimping’ students with difficult questions.^6^ Teachers may elevate their positions by behaving disrespectfully towards other professionals in students’ presence,^7^ and behaving insensitively towards patients and objectifying them during bedside teaching.^8^ Informal educational encounters,^9^ as well as formal teaching, influence social positioning because students ‘mimetically’ assimilate the people and practices they encounter into their own professional identities.^10^ Medical teachers’ cynical comments, for example, show students how to position themselves above others.^11^, ^12^ Doctors’ pro‐social behaviour, by contrast, can also contribute to professional identity formation. In an earlier study, some medical students identified with clinical educators who discursively narrowed the positional gap between themselves and patients to form therapeutic relationships. They rejected identities offered by clinicians who widened the gap by being aloof or behaving disrespectfully towards patients.^1^, ^3^ These observations suggest that understanding how medical teachers position doctors in relation to patients could contribute to the pedagogy of medical identity formation.^12^, ^13^

Social positioning was thoroughly explored by Holland and colleagues in their analysis of identity and agency in cultural worlds, known as ‘Figured Worlds’ theory.^4^ Holland et al^4^ drew on work by the discourse theorist Mikhail Bakhtin to give language a central place in social action and identity formation. People use ‘talk’ they have heard others use to ‘author’ identities. Talk offers social possibilities, from which people construct and reconstruct positions for themselves and others, in the moment, during the flow of everyday conversation. Over time, these relative positions become stable ‘dispositions.’

Translating these theoretical insights to the practice of medical education, the talk of a curriculum^14^ offers students a variety of positional identities in relation to patients. Talk does not have a deterministic effect on students’ identities, however, because students can respond individually to what they hear and see. They ‘author’ their own identities from the various positions offered to them by the many ‘figures’ they meet. This has important educational implications because it shows how the use of language can influence identity formation.^15^ Critical pedagogy has recently been proposed as a means of examining, critically, how language enacts discourses of power, privilege and position, and how these influence professional identity.^16^, ^17^, ^18^ The implementation of critical pedagogy in medical education, and thereby students’ identity formation, could be promoted by a clearer understanding of how curriculum talk influences social positioning.^19^, ^20^, ^21^

The purpose of this research was to help students develop identities that best serve the needs of patients.^3^, ^7^, ^12^, ^13^, ^14^ The research question was: How do medical teachers position doctors in relation to patients? We chose Figured Worlds theory and its companion qualitative methodology, dialogic analysis, to provide a robust methodological platform on which to answer the question.

## 2 METHODS

### 2.1 Ethics

The Joint Research Ethics Committee of the School of Medicine, Dentistry and Biomedical Sciences, Queen’s University Belfast approved the project (approval no. 15.19).

### 2.2 Setting

The research context was Queen’s University Belfast (QUB), a research‐intensive university, which had the only medical school in Northern Ireland, a geographically bounded region of the UK. Its 5‐year undergraduate medical programme provides 2 years of clinical science education with some clinical contact followed by 3 years in clinical settings.

### 2.3 Conceptual orientation

We chose a qualitative approach because this research set out to explore a complex social phenomenon. We took a critical realist position, which is well suited to exploring power relationships and the negotiation of identity.^22^ This position does not seek to reveal a ‘real’ world in the usual sense, but it allows researchers to use the critical analysis of language as a means of understanding social practices, institutions, structures and relationships.

More specifically, the analysis was informed by Bakhtin’s theory of dialogism, which is considered to have useful practical applications as well as being conceptually rich.^4^, ^22^ Dialogism takes the ontological position that people rely on others to develop a sense of who they are, but they also have a sense of their own creative potential. Speech is central to Bakhtinian thinking because it mediates sociocultural influences, the formation of individual identity, and the exercise of power. ‘Social speech’ is in continuity with ‘inner speech.’ In both the social and individual contexts, people are engaged in multiple dialogues. The ability to author the many voices they hear gives people social agency and individual identity. By contrast with, for example, cognitive theories, dialogic theory regards speech as the exchange of lived ideas, including personal values and judgements, rather than of purely abstract concepts. The concept of ‘addressivity’ has an important place in Bakhtinian scholarship. According to this concept, people are in a perpetual state of addressing others and being addressed by others. Building on Bakhtin’s work, Figured Worlds theory holds that, when we speak, we anticipate how the speech of others will author our identities and we author our own speech accordingly.

Dialogic methodology provides valuable possibilities for education research, which the distinction between ‘small‐d’ discourse and ‘Large‐D’ Discourse^15^ explains. Small‐d discourse is the everyday small talk by which the overt and hidden curricula position doctors and patients. Small talk reflects more stable and pervasive Large‐D Discourses, or dispositions, towards doctors’ social position. From this theoretical position, qualitative analysis of the small‐d language of curricula can uncover Large‐D Discourses. This theoretical approach is appealing because it has direct implications for curriculum development: educators could influence social positioning for the better by explaining it and encouraging students to take a critical stance towards what teachers say and do.

There is no single ‘correct’ way of actioning these theoretical insights, just as there is no single ‘correct’ interpretation of any one speech act. We chose to use Sullivan’s dialogic approach to guide this research.^22^ Sullivan proposed that researchers should regard language as being imbued with speakers’ intentions and desires, which are themselves entangled in social structures. Researchers using Sullivan’s methodology, approach qualitative analysis with several core assumptions. One is that speech is ‘language in action.’^15^ Another is that speech involves relations of power,^23^ and another is that language involves unconscious desire.^22^

### 2.4 Research team

Two QUB medical students (SRB and a classmate), who had completed 2 years of the programme, a former internist and education researcher working in QUB (TD), and an Irish family practitioner and education researcher now working in Canada (MK) conducted the research. Two were men and two were women. Their cultures of origin (from Singapore, Switzerland, the Republic of Ireland and England), as well as their ages and professional positions, provided varying sociocultural perspectives.

### 2.5 Design

This research explored how the language used by medical teachers in one‐to‐one, face to face interviews constructed doctors’ positions in relation to patients. The theoretical assumption that justified this design was that the social speech of a medical curriculum influences medical students’ inner speech. This influences students’ identity development, which influences how students position themselves in relation to patients.

### 2.6 Recruitment and participation

The researchers constructed a purposive sampling frame to ensure participants were scientists and clinicians of both sexes, who represented a range of ethnicities, career stages and disciplines. They included primary, secondary and tertiary care practitioners, all of whom contributed actively to the programme. Researchers invited medical teachers, by email, to contribute to an investigation into how medical teachers construct the position of doctors in relation to patients. They explained to prospective participants that the research was being conducted in order to help future medical students reflect critically on how they will position themselves in relation to patients after they qualify as doctors.

### 2.7 Data collection

**FIGURE 1:**
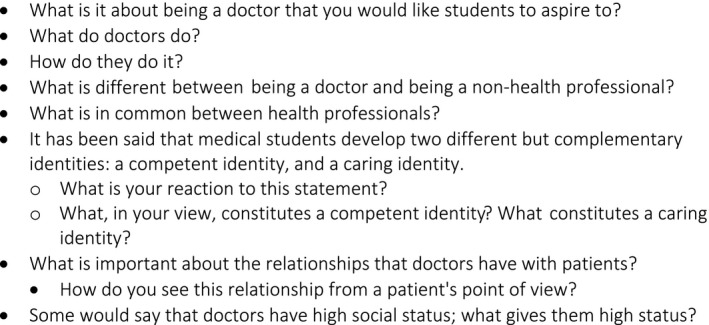
Interview prompts

Having weighed up whether students, teachers, or neutral third parties should interview participants, the team chose to use students because their relationship with participants most closely replicated the teacher‐to‐student conditions under which faculty members articulate the medical school’s discourses. During a summer elective, TD taught SRB and her fellow student‐researcher open‐ended exploratory interviewing by modelling and role‐playing it. TD listened to their recorded interviews and gave formative feedback on their interviewing techniques. The interviews used a deliberately open set of prompts (Figure 1) and interviewing techniques in order not to cue participants to author doctors’ positions in particular ways. The researchers audiorecorded the interviews, transcribed them verbatim, included key paralinguistic features (eg, pauses, laughter, hesitation), and pseudonymised them.

### 2.8 Data analysis

Sullivan’s dialogic approach requires researchers to arrive at their findings by achieving a balance between ‘bureaucratic’ and ‘charismatic’ approaches.^22^ A purely bureaucratic approach would vest authority in rules and procedures, systematic and exhaustive analysis of the transcripts, and researchers having an impersonal relationship with the data. A charismatic approach (for which no detailed guidelines exist) depends on researchers subjectively actualising the research procedures and responding reflexively to the data. We resolved possible tensions between these two extremes by conducting individual stages of data analysis meticulously, working reflexively as a team, and keeping an audit trail, when interpreting the data subjectively. We followed Sullivan’s advice not to conduct hierarchical coding, but to extract the speech acts (utterances) that were best able to answer the research question and ‘sound bites’ that epitomised participants’ different positions. We scrutinised the text for speech acts that exemplified variation amongst participants, contradictions within and between their discourses, and texts that gave doctors more or less status and power in different ways.

The interviewers, having each conducted and transcribed half the interviews, carefully read each other’s transcripts and TD read them all. They met to discuss preliminary interpretations and then independently analysed a single transcript in depth, as recommended by Sullivan,^22^ to identify discrete speech acts that answered the research question. Regular correspondence and face to face or emailed discussion helped the researchers question one another’s interpretations, which prevented any one perspective from dominating. Having closely agreed which speech acts answered the analytical question, we proceeded to the next stage of analysis.

TD, SRB and the second interviewer worked in pairs to read each transcript, identify relevant speech acts, interpret the texts and compare interpretations. Working individually, but regularly discussing their interpretations and asking one another’s opinions, they developed a template into which they pasted all 137 relevant speech acts (range: 8‐20 per participant) from all 10 transcripts, each accompanied by a free text memo explaining the researcher’s interpretation. Researchers copied blocks of text, rather than marking up words or phrases, to keep each piece of speech in its discursive context. They coupled these with analytical memos to make the process transparent to other team members. They elaborated the template and reapplied it to all speech acts. MK, who was naïve to the analysis up to that point, enhanced the team’s collective reflexivity by giving her independent opinion on a random 20% sample of speech acts.

This analysis identified seven main themes: what doctors do; what patients do; who doctors are; who patients are; changing times; social structures, and characteristics of the language. TD organised the data into these themes, which helped researchers identify four main discourses, described in the results section. To validate this provisional interpretation, the authors ran a workshop at an international conference, in which participants organised textual extracts into the four named discourses and gave critical feedback on the thematic structure. Then, the authors returned to the original audiorecordings to compare the interpretation against the data in their rawest state. Finally, they coded the data to the agreed themes using NVivo Version 12 analytic software (QSR International Pty Ltd, Doncaster, Vic, Australia) and wrote the narrative that follows using output from it.

Throughout these analytical stages (the details of which were recorded in a research log), researchers repeatedly re‐read individual participants’ speech acts, compared them against other participants’ speech, and sought patterns within and between individual participants’ responses.

### 2.9 Reporting

Dialogic analysis places considerable importance on how findings are reported, which should be in a manner that is rich in quotations and textual features derived from participants’ speech acts. The researchers’ authorial voice should assemble participants’ different voices into a coherent account of findings, highlighting and juxtaposing different positions.^22^ Consistent with dialogic epistemology, the final account should not be regarded as an incontrovertible statement of how medical teachers position doctors, but as an interpretation that can generate interest and stimulate debate. It would make the results unreadable if all fragments of speech acts were attributed to individual participants so we have done this (using pseudonym and speech act number) only for longer (italicised) quotations. We use the phraseology ‘patients were …, patients did …’ as shorthand for ‘participants’ discourses constructed patients as …’

## 3 RESULTS

Table 1 gives some details of the 10 participants but not their specific academic disciplines because this would make individuals too easily identifiable. Participants positioned doctors in relation to patients within discourses of Social Superiority, Technical Effectiveness, Benevolence, and Distributed Status and Responsibility (abbreviated to Superiority, Effectiveness, Benevolence and Distribution, respectively), which we now illustrate. The following quotation exemplifies how, in the Superiority Discourse, social class gives doctors a high social position:

It used to be that there’s the higher class, middle class and lower class and I think there were more lower class, you know. The less educated and the less financially well‐off people are, the higher your status. The higher you will be in their eyes. (G07)

**TABLE 1:** Details of participants

Pseudonym	Sex	Scientist or doctor	Career stage
GO	Female	Retired doctor	Senior
HM	Male	Doctor	Senior
JT	Female	Doctor	Junior
LJ	Male	Doctor	Mid
MO	Female	Doctor	Mid
OD	Female	Scientist	Mid
OY	Male	Doctor	Mid
TU	Male	Doctor	Senior
UT	Male	Scientist	Mid
UV	Female	Scientist	Junior

The Effectiveness Discourse invokes doctors’ mastery of technical solutions to define their position in relation to patients:

People can’t manage their own health. If they’re ill, they need the expertise … (UT4)

… the core defining thing is being able to deal technically or physically … with the patient’s illness. (OY4)

The Benevolence Discourse invokes doctors’ trustworthiness and empathy to construct doctors’ position:

The patient has to respect the doctor’s judgement and therefore trust is a big part … honesty, trust, respect … and empathy. (HM6)

By contrast with the Superiority, Effectiveness and Benevolence Discourses, the Distribution Discourse tends to narrow the distance between doctors’ and patients’ positions:

You … change your relationship with the patient depending on how the patient is and your relationship with the patient would be one of collaboration … okay let’s sit down [claps hands and rubs them together] and roll up our sleeves; what can we do about this? (UT7)

As the Superiority, Effectiveness, and Benevolence Discourses tend to widen the gap between patients’ and doctors’ positions, we first cluster these three Discourses and explain how they construct first patients’ and then doctors’ positions. We then describe tensions associated with these discursive positions. Finally, we present the contrasting Distribution Discourse.

### 3.1 Positions constructed by the Superiority, Effectiveness and Benevolence Discourses

#### 3.1.1 Patients’ positions

Patients, as constructed by the Superiority, Effectiveness and Benevolence Discourses, are sick, fearful, vulnerable and requiring of help from doctors, to whom they look up and give respect. Patients ‘*come in through the door*’ (LJ3) for guidance, help and advice. They wait their turn, tell ‘*doctors their deepest darkest*’ (UT6) and then go away. They ‘*expect doctors to be of the utmost integrity*’ (UV5), to tell the truth, keep secrets, and behave according to professional standards. Patients expect doctors to ‘*do their very best to help … [and not] … dismiss you and say … oh, well you shouldn’t be worried about that*’ (UV11). Patients expect doctors ‘*to listen to the patient’s concerns and take time and not rush you*’ (UV11). These expectations place a burden on doctors.

Adjectives like ‘vulnerable’ and phrases like ‘looking up’ and ‘expecting not to be dismissed’ give patients low positions. Participants’ language subordinated patients in other ways. It used the grammar of possession to describe relationships, such as ‘*if your patient is having a heart attack*’ (GO6) and ‘*you’ve got an old man who’s confused, thrashing about the bed*’ (TU12). Metaphors give patients the lowly status of commodities, such as in: ‘dealing’ with patients or ‘building up’ cases. Language widens the gap between the positions of patients and doctors in more direct ways: patients do not always want doctors ‘*to leave it too much to the actual patient*’ (MO8). ‘*Patients … nearly want the doctor to be all‐powerful so they could be sorted out*’ (MO14). Language also distances doctors from the consequences of patients’ actions such as when they ‘*haven’t complied with the treatment*’ (OD9). Patients are ‘*responsible for their own health until they walk back in the door*’ (OD12).

There are differences as well as similarities between the Superiority, Effectiveness and Benevolence Discourses. The phrase ‘*put you on a pedestal*’ (GO1), which characterises Superiority, uses a grammatical construction that elevates doctors and the evocative metaphor of a pedestal to emphasise the doctor’s position. The language of the Effectiveness Discourse, which describes patients as coming to doctors to be ‘sorted,’ creates distance by giving doctors the agency to ‘sort’ and patients no more agency than that required to come to the doctor. The Benevolence Discourse creates distance in a way that is epitomised by the phrase ‘*a doctor is someone that people will want to put their trust in*’ (UV7). Doctors’ trustworthiness merits investment, whereas patients have no more agency than to invest in doctors.

#### 3.1.2 Doctors’ positions

Participants’ use of religious and family metaphors elevated the position of doctors. Doctors are *nearly god‐like* or like *priests*. Their words are ‘gospel’:

I mean when … you’re worried about dying … this talk about vocation like nearly is a religious … I think some people want doctors to be all powerful. You go along with all your problems and they will sort them out. Like they’re a mother or father or their priest or whatever. (MO14)

Military metaphors also elevate doctors’ position: ‘*being the knight in shining armour … is very empowering … very fulfilling and makes you feel very useful as a person*’ (UT2). Surgeons could ‘*have a big impact on people’s health*’ (UV8). Having ‘people skills,’ knowing what to say and when to say it, and being a good communicator elevate doctors’ positions, as do metaphors of commodity trading. By seeing many patients, doctors build expertise. They deal with patients, their illnesses and their life issues. They sort out patients’ problems. They handle adversity, unexpected events and problematic situations. They deliver care. They give time and information. These transactions make life‐changing differences.

##### Superiority

Constructions such as being *held in high regard*, being *perceived as a very valuable member of society* and being *cornered into … being the authority figure* exemplify how participants grammatically constructed doctors’ high status.

##### Effectiveness

Doctors have elevated positions in the Effectiveness Discourse because they have a ‘*degree of training and education … [that] automatically puts [them] in a position of power because [they] know more than the patient*’ (OY19). This Discourse tends to elevate technical over emotional aspects of practice:

… you have to develop a certain distance in order to function effectively. […] Callous behaviour is a fairly superficial behaviour that may shock non‐health care professionals but underlying it people still have a caring nature. (OY8)

Being competent allows doctors to:

… deal with the medical side of people’s illnesses … Knowing what drug to give and when to give it and what dose to give. Knowing … where to make the cuts, to … start a heart bypass operation. (OY9)

There was a ‘*buzz of having to do something difficult and having to deal with people who are maybe very badly hurt in say a trauma situation*’ (TU2).

A characteristic grammatical feature of the Effectiveness discourse is the use of action verbs. Doctors ‘*got … complicated case(s)*’ (UV4), which they ‘*medically managed* by *taking histories*, *making diagnoses*, *giving advice*, *giving drugs*, *operating*, *referring* and *curing* ' (UV4). The following quotation elevates doctors’ position by emphasising the effectiveness of technical solutions:

And the surgeon [laughs] he just diagnosed it was renal colic and it was kidney stones because it was so excruciatingly painful. And the first thing you do … is give them an injection of pethidine … And then give them another if that first one isn’t strong enough, and then they just love you [laughs]. (GO4)

Doctors’ mastery of effective technologies raises their position:

But actually you know they [patients] come in looking for advice and I probably end up following quite a paternalistic mode. But I try and inform them, you know I guess, so fine, I’m the authority figure, I’m the expert … I’m telling them what treatment they’ll need. (OY13)

Doctors ‘*tried to get someone to manage their diabetes effectively*’ (OD9), ‘gave *straight down the line instructions*’ (UT7), and ‘*got people to do what you want whether it’s take their [treatment] or run a clinic for you the way they want*’ (OY2).

##### Benevolence

Doctors, in the Benevolence Discourse, have ‘*a real desire to help … and to make a difference*’ (OY12). Their position is elevated by caring about patients and being, as a result, role models and mentors. They ‘*put the needs of the patients first, uppermost.’ They are ‘able to empathise with the patient ' (OY12). Although they ‘want to … empower the patient, they also have an idea of what’s going to be in the patient’s interest’ (HM4). When a patient ‘turns around and says, doctor, doctor, am I going to die? I look at you straight in the eye and I give you a gentle but an honest answer, I won’t tell you lies’ (TU16)*.

### 3.2 Challenges to superiority, effectiveness and benevolence

There were many references to how doctors’ position is becoming less elevated. One cause was redistribution of wealth. Another was wider availability of education. Another was travel. The main one was availability of knowledge:

More knowledge, definitely more knowledge. Medicine’s not a mystery anymore. You can Google it. There’s medical programmes on TV, people have more life experience, people are travelling and people just know more about everything. (GO8)

Resistance to these challenges was apparent in participants’ responses, evidence for which is presented in Table 2. One participant described doctors’ lessened status as a ‘deterioration’:

Many years ago, doctors were held in very high esteem … maybe that’s deteriorated down through the years. (GO1)

**TABLE 2:** Linguistic evidence of resistance

Phrase or comment	Linguistic feature	Position defended by this language
*‘Sometimes it is lovely when people don’t know that you are medical because they maybe put you on a pedestal and they don’t treat you the same way they would treat a normal … you’re not a normal human being to them’* (GO10)	Inherent contradiction between doctors as normal or abnormal	Doctors’ social status
*‘The thing that I like about medicine and dealing with patients is that you have to be communist about it’* (OY17)	Contradiction between ‘dealing with patients’ and ‘being communist’	Doctors’ social status
*‘They will know that they will, should be able to have a relationship that they can challenge, the decision from the doctor, and uh … and … maybe have to see a different doctor for example’* (LJ8)	Hedging	Doctors’ expertise
*‘It’s quite refreshing when a patient comes in and has questions and is challenging things but you just wouldn’t have time for that’* (OY14)	‘But’; negation	Doctors’ expertise
*‘I’m telling them what treatment they’ll need but just to try to make a chink into getting them involved I show them their scans’* (OY13)	‘Chink’; metaphor	Doctors’ expertise
*‘For the 20 minutes I’m in front of them I’ve a real desire to help them and to make a difference but once they walk out of the room, yes I still care for them but I just don’t have the capacity to think about them once they walk out the door. They’re sorted and they’re responsible for their own health until they walk back in the door’* (OY12)	‘But’; negation	Doctors’ expertise
*‘I said that the relationship that we had after the [second world] war would have been doctor, whatever you think, whatever, you know best, doctor. So now that relationship is [sigh] not, not always, don’t leave me with the impression that it’s all difficult, it’s not all difficult, most of the time it’s okay. It’s more of a shifting sands relationship. So I’m much more, if I’m a patient now and I’m coming to see you in your generation in 10 years’ time for example, I’ll be, I’m full of knowledge, I’ve been on the Internet, I’ve got a massive amount of knowledge. I will have gone on to my patient support group for example to find the questions to ask you, and furthermore I’ll not be content maybe with your own opinion, I might want a second opinion and a third opinion’* (TU17)	‘Shifting sands’ relationship	Doctors’ social status and knowledge

A participant’s use of words to describe how patients choose between doctors implied resistance:

You never know if somebody comes to you for your advice, or they’re away off to another consultant. (GO7)

Another participant contrasted ‘publicly available knowledge’ unfavourably with evidence:

… the patient comes pretty well informed, they may not have the evidence base, the doctor will have the evidence base to support the various treatments, but the patient won’t have that, they just have maybe what somebody in a chatroom on Google had suggested was an appropriate type of treatment. (OD10)

Another participant said patients were opting for paternalism:

… patients invariably say, ‘I don’t know, it’s up to you doc, you decide.’ So patients seem to be reverting to a paternalistic mode of medicine. (OY13)

Another participant dismissed ‘absolutely empowered’ patients’ mistrust of doctors:

… that old‐fashioned trust is a bit more now tempered with, I might get a second opinion, I might get a third opinion, and if I don’t get on well with you, I’ll go somewhere else, and I’m not afraid to go somewhere else, because I am now empowered, I … am empowered, absolutely empowered by knowledge. (TU17)

### 3.3 Positions constructed by distribution

The Distribution Discourse was less often articulated, but clearly present and different from the Superiority, Effectiveness and Benevolence Discourses. The following quotation exemplifies how doctors, according to this Discourse, could actively distribute status and responsibility by increasing patients’ agency:

… being a doctor the less I see as trying to solve problems or fix problems, it’s kind of a chance to be with people trying to enable people to be the best people that they can be. (JT1)

Participants’ use of verbs distributed power: Doctors ‘*work on … shared decision‐making*’ (JT2); they ‘*signpost; fulfill the role of social worker cum benefits adviser*’ (JT3); ‘*help people do things; tailor their work to the needs of individuals [and] discuss*’ (OD5). Doctors’ identity is ‘*as a non‐judgemental advocate for the patient*’ (OD7). Doctors’ work is ‘*a team effort [in which] these are the things that you have to do for yourself and these are the ways I can help and support you*’ (OD8). Doctors ‘*use their power to enhance health*’ (OD15). Distribution is a discourse of negotiation: ‘*what are you prepared to do, are you prepared to change your diet? We have this drug, that drug or the other drug, you know*’ (UT7). Doctors ‘*try to be … facilitative in establishing … what has gone wrong here … instead of challenging them you have to build up their trust to get them to be honest with you*’ (OD9).

Whereas doctors took the initiative to distribute status and responsibility in the preceding quotations, the next excerpts show how patients could, also, redistribute: patients ‘*might perceive us as wielders of more power than we perhaps are … you stopped my benefits … as if we hold more authority over … aspects … that affect their lives (more) than we actually do*’ (JT9). Patients see doctors as ‘*wielders of power and holders of the magic tickets and … way of accessing things that they need … giving them money … giving the drugs they need*’ (JT9).

## 4 DISCUSSION

The answer to our question ‘How do medical teachers position doctors in relation to patients?’ is that the Superiority, Effectiveness and Benevolence Discourses give doctors elevated social positions by virtue of their social status, knowledge and technical skills, and trustworthiness, respectively. These discourses were dominant^23^ in two senses: they were the most commonly articulated discourses; and they positioned doctors as more socially elevated than patients. However, the force of social change was in tension with these Discourses, as evidenced by participants’ self‐authored defence against change: doctors’ less elevated position nowadays was a ‘deterioration’; ‘somebody in a chatroom on Google’ had, implicitly, less authority than ‘the evidence base’; ‘absolutely empowered patients’ were getting second and third opinions because they didn’t ‘get on well with you,’ and other patients were ‘reverting to a paternalistic type of medicine.’ Whereas the three dominant Discourses defend doctors’ elevated position against challenges to their status, and privilege access to knowledge and trustworthiness, the non‐dominant Distribution Discourse narrows the gap between doctors’ and patients’ social positions: doctors ‘use their power to empower patients,’ and when patients demand what is not available, ‘doctors negotiate solutions.’ Balancing imbalances of power and status was seen as intrinsic to practice.

These findings support and elaborate on the limited research to date on social positioning in medical education, reviewed earlier. We have confirmed that, within the Superiority, Effectiveness and Benevolence Discourses, illness lowers people’s social positions.^1^, ^4^, ^5^ We have confirmed that, within the Distribution Discourse, doctors redistribute power to narrow the positional gap, thereby elevating ill people’s positions.^2^, ^3^ To this we have added a thick description of four Discourses, which we recognise, reflexively, as prevalent in the medical profession and believe are capable of promoting critical reflection in medical education. Our work adds to a small, but growing, body of research into the cultural shaping of students’ and doctors’ positions and identities.^24^ Students discursively assume the social position of doctors‐to‐be when they start interacting with patients.^24^ They negotiate tensions between the imagined position of doctors, who have the power to ‘make a difference’, and the experienced position of students, who are powerless.^25^ The positions that students’ adopt and the positions they give patients and doctors differ between culturally different parts of the world.^26^ Students have been shown to identify with positions taken by doctors who behave humanely towards patients and to reject positions taken by inhumane doctors.^3^ Women find there is little ‘discursive space’ for them in the male‐dominated world of surgery and adopt masculine positional identities as a result.^27^ Novice clinical teachers have to navigate and reconcile different subject positions in order to develop the identity of a teacher.^28^ Taken as a whole, this body of research suggests that identity positioning is a fruitful field of medical education research.

Dialogical research has inherent limitations. It uses theory to support a deliberately critical stance. It acknowledges that other methodologies may interpret the same data differently. Rather than purporting to describe reality, dialogical research examines familiar situations in revealing new ways. This allows researchers to offer tentative interpretations, which are not to be judged so much for their accuracy as for their ability to provoke socially relevant debate. There is an important difference between being critical and being judgemental. Our findings are interpretations of spoken words, which seek to analyse social position, but make no claims to analyse participants’ thoughts or actions. Our findings reflect the culture in which the research was conducted, which limits their transferability. Analysing interviews rather than analysing informal speech may have affected the discourses in unknown ways. The results may have been different if the interviewers had not been medical students, although there is no way of knowing if that would have made them more valid. They might, also, have been different if the reviewers had used different prompts in the interviews. To reduce any undue influence of the questions on the findings, we selected text that was not a direct response to interviewers’ cues, and used non‐directive prompting to probe participants’ responses. The one exception was the final question, which gave doctors a position, but used a language construction that implicitly invited participants to agree or disagree with the suggestion made.

The implications of this work for medical education link with another emerging research topic, critical pedagogy. The purpose of this is to help students develop critical consciousness of doctors’ and patients’ social positions so that they develop the identity of compassionate, humanistic, socially conscious health professionals.^17^, ^18^, ^29^, ^30^ We offer our framing of doctors’ positional relationships with patients as subject matter for critical pedagogy. We hope this will help students reflect on how doctors can use their position, and the power that comes with it, in virtuous ways rather than the vicious ways they may sometimes observe.

## 5 CONCLUSIONS

We ask students, educators and doctors to reflect on how discourses of social superiority, technical effectiveness, benevolence and the distribution of power and responsibility, influence their relationships and other people’s relationships with patients, so that we may all use our positions to best effect.

## AUTHOR CONTRIBUTIONS

TD conceived the idea for this study, supervised the project, analysed the data, and wrote the article. SRB conducted the fieldwork, participated in the analysis, and contributed to the final manuscript. MK participated in the analysis and contributed to the final manuscript.

## CONFLICTS OF INTEREST

None.

## ETHICAL APPROVAL

The Joint Research Ethics Committee of the School of Medicine, Dentistry and Biomedical Sciences, Queen’s University Belfast approved the project (approval no. 15.19).
